# Epigenetic activation of LY6K predicts the presence of metastasis and poor prognosis in breast carcinoma

**DOI:** 10.18632/oncotarget.10972

**Published:** 2016-08-01

**Authors:** Hyun Kyung Kong, Sae Jeong Park, Ye Sol Kim, Kyoung Min Kim, Hyun-Woo Lee, Hyeok-Gu Kang, Yu Mi Woo, Eun Young Park, Je Yeong Ko, Hiromu Suzuki, Kyung-Hee Chun, Erwei Song, Kyu Yun Jang, Jong Hoon Park

**Affiliations:** ^1^ Department of Biological Science, Sookmyung Women's University, Seoul, Republic of Korea; ^2^ Department of Pathology, Chonbuk National University Medical School, Research Institute of Clinical Medicine and Research Institute for Endocrine Sciences, Jeonju, Republic of Korea; ^3^ Department of Biochemistry and Molecular Biology, Yonsei University College of Medicine, Seoul, Republic of Korea; ^4^ Department of Biochemistry, College of Life Science and Biotechnology, Yonsei University, Seoul, Republic of Korea; ^5^ Brain Korea 21 PLUS Project for Medical Science, Yonsei University College of Medicine, Seoul, Republic of Korea; ^6^ Department of Molecular Biology, Sapporo Medical University, Sapporo, Japan; ^7^ Department of Breast Surgery, Sun Yat-Sen Memorial Hospital, Sun-Yat-Sen University, Guangzhou, Peoples Republic of China

**Keywords:** breast cancer, LY6K, metastasis, DNA methylation, histone modification

## Abstract

The role of lymphocyte antigen 6 complex, locus K (LY6K) in breast cancer has been studied, whereas the epigenetic control of LY6K transcription is not fully understood. Here, we report that breast cancer patients with increased LY6K expression had shorter disease-free and overall survival than the patients with low levels of LY6K by multivariate analysis. LY6K also was upregulated in breast cancer patients with distant metastases than those without distant metastases, downregulating E-cadherin expression. Furthermore, xenograft tumor volumes from LY6K knockdown nude mice were reduced than those of mice treated with control lentivirus. Interestingly, LY6K has a CpG island (CGI) around the transcription start site and non-CGI in its promoter, called a CGI shore. LY6K expression was inversely correlated with methylation in not only CGI but CGI shore, which are associated with histone modifications. Additionally, LY6K methylation was increased by the PAX3 transcription factor due to the SNP242 mutation in LY6K CGI shore. Taken together, breast cancer risk and metastasis were significantly associated with not only LY6K expression, but also methylation of CGI shore which induced by SNP242 mutation. Our results suggest that an understanding epigenetic mechanism of the LY6K gene may be useful to diagnose carcinogenic risk and predict outcomes of patients with metastatic breast cancer.

## INTRODUCTION

The relationship between breast cancer and epigenetics was first revealed in 1983 [1]. Over the past few years, there has been an exponential increase in breast cancer epigenetic studies [2]. Epigenetic tools are emerging as a new diagnostic and therapy target to treat breast cancer. Detection of breast cancer at an early stage is the key to successful treatment and patient outcomes [3]. Considering the benefits of epigenetics, a change in DNA methylation may be regarded as a useful biomarker of breast cancer because, compared to other sources such as mRNA and protein, DNA is relatively stable and can be obtained as cell-free DNA from the blood, ductal lavage fluids, nipple aspirate fluids, and fine needle aspirates of primary tumors [4–8]. A number of studies have reported the ability to detect breast cancer cells by epigenetic analysis in fine needle aspirations, nipple aspirates, and ductal lavages [9–12]. Therefore, epigenetic alterations such as specific gene DNA methylation, histone modifications, or other events hold promise as tools for early detection of breast cancer.

The invasiveness and metastatic function of LY6K in cancer as a diagnostic biomarker and therapeutic target has been elucidated in various cancers such as head and neck squamous cell carcinoma, breast cancer, lung and esophageal carcinomas, bladder cancer, and esophageal squamous cell carcinoma [13–18]. Additionally, LY6K is a target for cancer vaccine therapies because stimulating cytotoxic T lymphocytes by endogenously expressed LY6K presents a specific cytotoxic activity against lung and esophageal squamous cell carcinoma [19]. Therefore, targeting LY6K epigenetic modification may be a useful cancer therapeutic strategy.

In this report, we investigated the relationship between chromatin modifications and their association with DNA methylation patterns and LY6K expression in breast carcinoma and cell lines. First, we found that LY6K expression was significantly correlated with overall survival (OS) and distant metastasis, while DNA methylation was inversely associated with LY6K expression in breast cancer cell lines and tumors. In addition, xenograft tumors were reduced in nude mice that downregulated LY6K following lentivirus treatment. Interestingly, methylation of LY6K non-CGI promoter was increased by PAX3 due to the SNP242 mutation. These observations highlight a novel mechanism of LY6K methylation and expression. Finally, this study proposes understanding of epigenetic changes in LY6K may contribute to the diagnosis of cancer risk and prognosis of patients with breast cancer.

## RESULTS

### LY6K as an independent prognostic maker for metastasis and overall survival in patients with breast carcinoma

**Figure 1:**
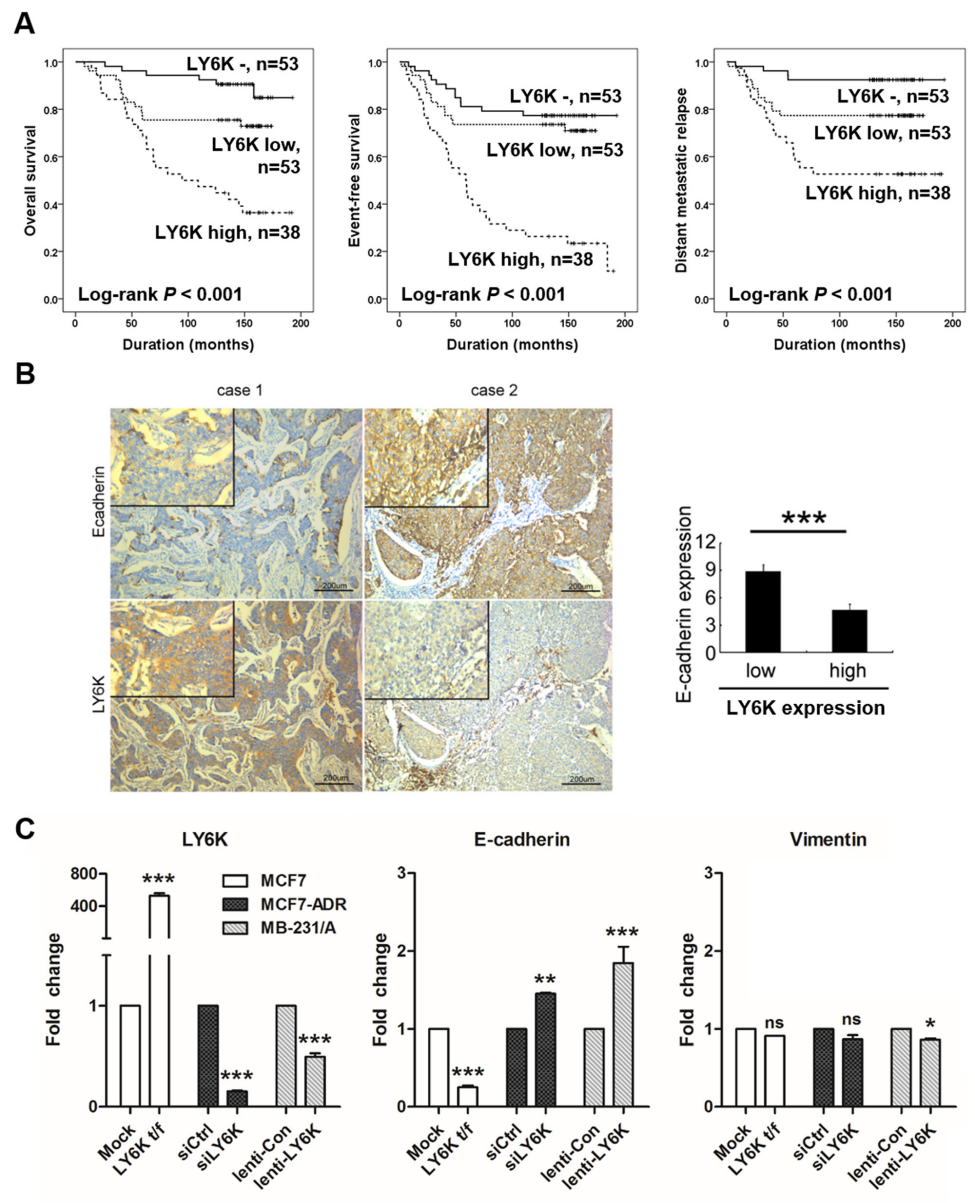
Kaplan-Meier survival analysis and LY6K induces metastasis by regulating E-cadherin **A.** Overall survival, event-free survival, and distant metastatic relapse according to the expression of LY6K in 144 breast carcinoma. **B.** E-cadherin was inversely correlated with LY6K expression in tumor. **C.** mRNA expression of epithelial (E-cadherin) and mesenchymal (vimentin) markers were determined in breast cancer cell lines transfected with LY6K or siRNA or lenti-viral particle. LY6K was normalized by 18s rRNA. All graphs show mean ± SD (error bars) of independent experiments. *, *P* < 0.05, **, *P* < 0.001, and ***, *P* < 0.0001.

LY6K immunohistochemical analysis using 144 clinical samples indicated that LY6K mainly localized in the cell membrane and cytoplasm of the tumor cells, while peri-tumoral stromal tissue did not expressed LY6K (Supplementary Figure S1). LY6K expression significantly associated with older patients (*P* = 0.006) and latent metastasis at the distant site (*P* < 0.001) (Supplementary Table S2). In the univariate and multivariate Cox model, TNM stage, HER2 expression, and LY6K expression were significantly associated with OS, EFS, and DMR (Supplementary Table S3 and Table 1). In addition, we analyzed the prognostic significance of LY6K expression in the subgroups of patients who received adjuvant chemotherapy or endocrine therapy, and ER-positivity of the tumors (Supplementary Figure S2). Higher levels of LY6K without reference to subgroups were independently associated with greater risk of death, shorter EFS, and greater risk of distant metastatic relapse when compared with patients with LY6K-negative tumors (Figure 1A).

**Table 1:** Multivariate Cox regression analysis for overall survival and event-free survival in breast carcinoma patients

Characteristics		OS		EFS		DMR	
		HR (95% CI)	*p*-value	HR (95% CI)	*p*-value	HR (95% CI)	*p*-value
TNM stage	I	1	0.021			1	0.056
	II	2.853 (0.997-8.166)	0.051			1.605 (0.552-4.664)	0.385
	III and IV	5.138 (1.601-16.485)	0.006			3.696 (1.111-12.297)	0.033
HER2	negative	1	0.002	1	0.003		
	positive	2.664 (1.443-4.919)		2.242 (1.307-3.845)			
LY6K	negative	1	< 0.001	1	< 0.001	1	< 0.001
	low	2.158 (0.820-5.678)	0.119	1.187 (0.552-2.554)	0.661	3.349 (1.079-10.391)	0.036
	high	6.333 (2.565-15.641)	< 0.001	4.449 (2.256-8.772)	< 0.001	7.597 (2.564-22.505)	< 0.001

Abbreviations: OS, overall survival; EFS, event-free survival; DMR, distant metastatic relapse; HR, hazard ratio; 95% CI, 95% confidence interval; HER2, human epidermal growth factor receptor 2.

### LY6K reduces E-cadherin expression in breast carcinoma and cancer cell lines

When we further analyzed this set of clinical samples, we found that LY6K-positive breast cancer tissues presented a lower E-cadherin, an epithelial marker, expression level, but E-cadherin expression increased in LY6K-negative breast tumor samples (Figure 1B). We investigated the relationship between the expression of these EMT-related markers and LY6K in breast cancer cell lines. Interestingly, we found that E-cadherin expression level significantly decreased in LY6K overexpressing cells but did not have an effect on vimentin, a mesenchymal marker (Figure 1C). Seven of the 12 human breast carcinoma cell lines (58%), MCF7-ADR, MDA-MB-157, MDA-MB-231, MDA-MB-435, MDA-MB-436, MDA-MB-468 and SK-BR-3 lines expressed the highest level of LY6K mRNA (Supplementary Figure S3). In contrast, MCF7, MDA-MB-361, MDA-MB-453, T47D, and ZR-75-1 cell lines did not express LY6K mRNA (Supplementary Figure S3). We transiently and stably knocked down LY6K in the LY6K-positive breast cancer cell lines, MCF7-ADR and mouse adapted MDA-MB-231 cells (MB-231/A), using siRNA and lentivirus, respectively. We found that E-cadherin expression was induced by LY6K knockdown but vimentin expression was not decreased by LY6K knockdown in both MCF7-ADR and mouse adapted MDA-MB-231 cells (Figure 1C).

### Stable knockdown of LY6K inhibited the growth of breast cancer xenograft in nude mice

**Figure 2:**
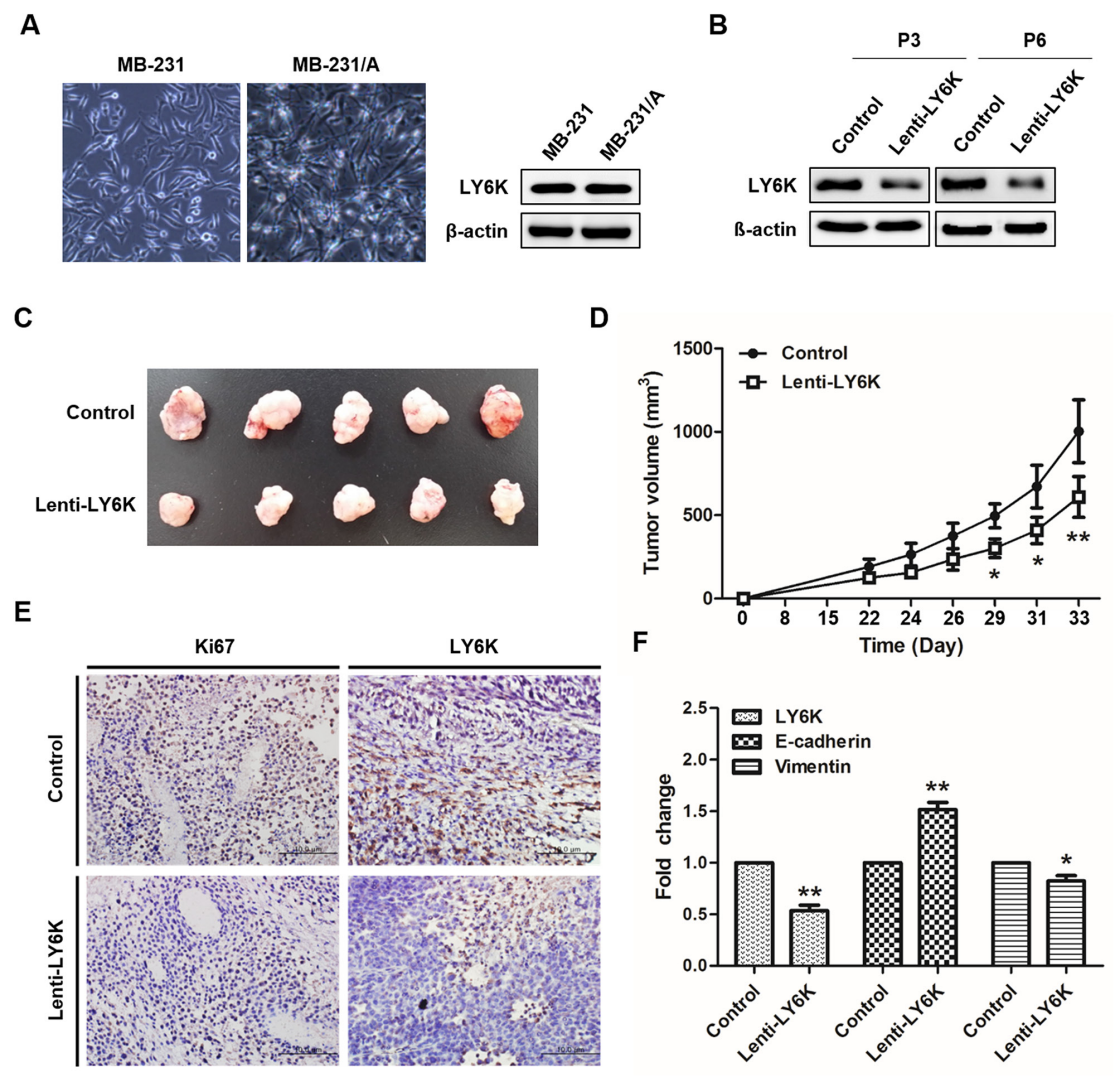
Tumorigenesis effect of LY6K *in vivo* **A.** Cellular morphology of mouse adapted MB-231 cells and protein expression level of LY6K. **B.** Downregulation of LY6K protein was confirmed in passage 3 and 6 treated with LY6K-lentivirus. **C.** Inhibition of the growth of breast cancer xenograft in nude mice by lentiviral transduction of LY6K. **D.** The size of tumors arising from LY6K stably downregulating was significantly lower than those of the control group at 33 days after inoculation. **E.** Immunohistochemistry analysis of lenti-LY6K tumor was inversely correlated with Ki67 as a marker of cell proliferation. (magnification, × 200; scale bar, 100 μm). **F.** LY6K expression was decreased in lenti-LY6K tumors (n=5), whereas E-cadherin increase compared with tumors of control (n=5). *, *P* < 0.05, and **, *P* < 0.001 significantly different from respective control group.

We next determined the effect of LY6K on the growth breast cancer xenograft in nude mice using mouse adapted MDA-MB-231 cells, called MB-231/A. Cancer cell populations can more survive in secondary xenograft assay than primary xenograft [20]. As shown in Figure 2A mouse adapted MDA-MB-231 cells shows more sharp-shaped than MB-231. LY6K expression level was stably knockdown in lentivirus-treated cells (Figure 2B). 1 × 10^6^ stable cells were injected into nude mice to establish secondary xenograft. At day 33 after injection, lentivirus-treated stable knockdown of LY6K tumors were smaller and grew more slowly as compared with respective control tumors transduced with empty lentiviral particles (Figures 2C and 2D). Immunohistochemistry staining revealed that LY6K showed lower levels and the Ki-67 positive cells was significantly reduced in LY6K lentivirus-treated xenografts (Figure 2E). Also E-cadherin still showed significantly higher levels in MB-231/A xenografts of the stable LY6K downregulating group (Figure 2F).

### Detection of LY6K methylation in surgical breast cancer samples and breast cancer cell lines

**Figure 3:**
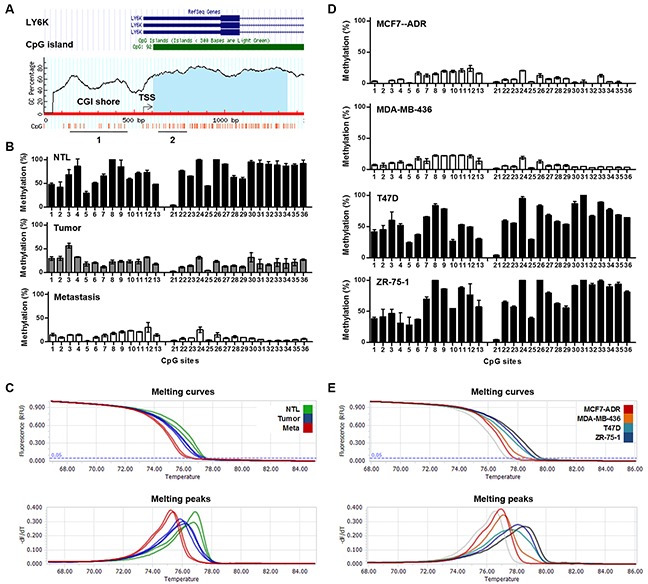
Methylation of LY6K in breast tumors and cancer cell lines **A.** Schematic map of LY6K showing the location of the CpG sites, transcription start site, and primers used for BSP (1 and 2), MCA (1) and MSP (1 and 2). **B.** and **D.** Bisulfite analysis from NTL, breast tumors and metastatic tumors (B) and breast cancer cell lines (D). **C.** and **E.** Melting curves and derivative peaks from four from NTL, breast tumors and metastatic tumors (C) and cancer cell lines (E).

We used the UCSC genome browser and the online Methprimer software to identify CGI in the region around the transcription start site of the LY6K (Figure 3A). MCA was performed to assess LY6K methylation in CGI shore and bisulfite pyrosequencing were performed to confirm the results with selected breast tissues and cell lines in both CGI and CGI shore regions. An excellent concordance between the results obtained by bisulfite pyrosequencing and MCA methods was observed. LY6K methylation was analyzed by MCA in 30 breast carcinomas and 15 NTL breast tissues. On the basis of BSP, NTL, tumor and metastatic tumors had 71%, 22% and 11% of 29 CpG sites methylated especially CGI shore region had 64%, 26% and 16% of 13 CpG sites methylated, respectively, whereas the estimated percentage of methylation by MCA was 69%, 35% and 19% (Figures 3B and 3C). The concordance was also breast cancer cell lines with BSP methylation of 13 CpG sites within CGI shore demonstrating 18% and 21% in LY6K-positive MCF7-ADR and MB-436 cells, and 60% and 71% in LY6K-negative T47D and ZR-75-1 cells, whereas the estimated percentages of methylation determined by MCA were 14%, 26%, 68% and 85%, respectively (Figures 3D and 3E). Finally, we compared LY6K expression levels and methylation status in breast cancer subtypes using the public datasets obtained from cBioPortal TCGA database (mRNA microarray and methylation array HM27) [21, 22]. LY6K mRNA expression was higher in triple-negative breast cancer subtype (TNBC) as compared with other subtypes (Supplementary Figure S5A). Interestingly, LY6K methylation and expression were significantly negatively correlated not only in TNBC (Pearson's *P* value = −0.683) but also other subtypes (Supplementary Figure S5B).

### SNP242 mutation increases DNA methylation which suppresses LY6K expression

**Figure 4:**
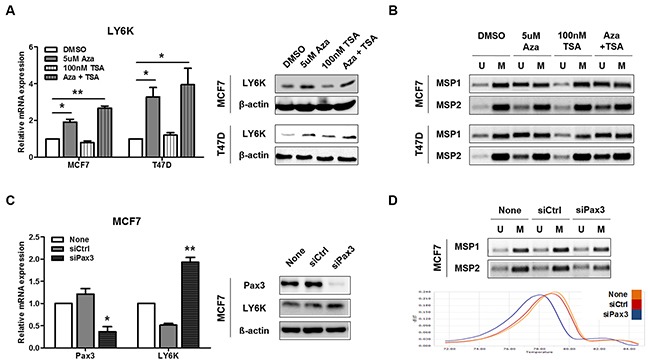
Pax3 inhibits LY6K expression by regulating methylation **A.** LY6K expression was analyzed by qRT-PCR and western blotting before and after stimulating the MCF7 and T47D cell lines with 5-aza-dC and/or TSA. 18s rRNA and β-actin expression were analyzed as controls. **B.** MSP analysis of the LY6K CGI shore and CGI in MCF7 and T47D cell lines before and after stimulation with 5-aza-dC and/or TSA. **C.** Expression level of Pax3 and LY6K after siRNA transfection normalized with 18s rRNA. **D.** MSP analysis and melting curves from Pax3 siRNA treated cell lines. All graphs show mean ± SD (error bars) of three independent experiments. *, *P* < 0.05, and **, *P* < 0.001.

LY6K mRNA and protein expression were markedly increased after 5 μM 5-aza-dC for 72 h, but not after TSA treatment, in the hypermethylated LY6K-negative cell lines MCF7 and T47D (Figure 4A). When MCF7 were treated with 5-aza-dC, MSP showed reduced intensity of the methylated DNA bands in comparison to that in the untreated cell lines, which paralleled the increase in amplification products when primers for unmethylated DNA were used (Figure 4B). Some breast carcinomas and cell lines expressed LY6K, but others did not [23]. LY6K-negative cells presented a C to G SNP242 substitution on LY6K promoter that creates *de novo* PAX3 binding sites therefore PAX3 transcription factor directly bind to LY6K promoter of mutated SNP242, which influence AP-1 binding affinity and reduce LY6K expression [23]. PAX3 is known to repress transcription by selectively binding with heterochromatin protein (HP1) and KAP1, which makes closed chromatin [24]. We hypothesized that PAX3 transcription factor binding, due to the SNP242 substitution, might increase LY6K promoter methylation and reduce AP-1 binding and LY6K expression. To confirm that PAX3 increases DNA methylation on LY6K promoter in LY6K-negative MCF7 cells, we analyzed DNA methylation status and expression of LY6K after treatment with a PAX3 siRNA. PAX3 siRNA treatment, the expression of LY6K was restored and methylation determined by MCA was shifted to the left compared with control, indicating that LY6K promoter was activated by PAX3 siRNA through a reduction in methylation (Figures 4C and 4D).

### Distinct histone modifications in the LY6K CGI shore correlate with LY6K expression status

**Figure 5:**
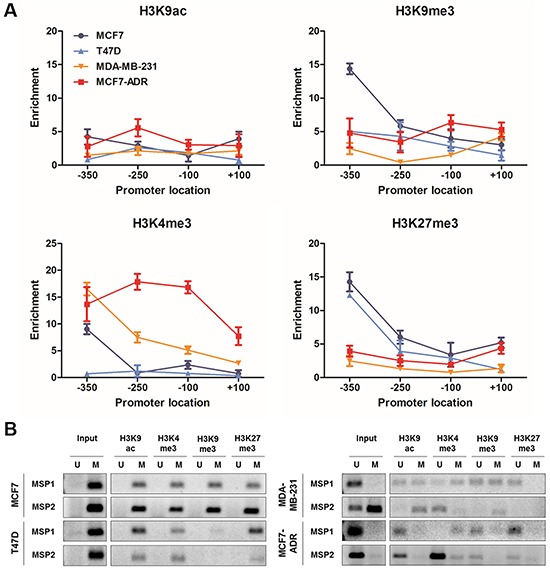
Histone modification and associated-DNA methylation patterns of LY6K **A.** ChIP qPCR was performed using the four primer sets listed in Supplementary Table S1 in MCF7, T47D, MCF7-ADR and MDA-MB-231 cell lines. Occupancy of each of the chromatin marks: acetylation of histone H3K9 (H3K9ac), trimethylation of histone H3K4 (H3K4me3), trimethylation of histone H3K9 (H3K9me3) and trimethylation of histone H3K27 (H3K4me3). **B.** ChIP-MSP analysis around LY6K transcription start site in MCF7, T47D, MCF7-ADR and MDA-MB-231 cells using MSP1 and MSP2 primers.

We performed a ChIP-qPCR analysis of the histone tail modifications using several primer sets spanning the region of interest and antibodies for both transcriptionally active (acH3K9 and H3K4me3) and inactive (H3K9me3 and H3K27me3) marks of chromatin to characterize the chromatin architecture associated with the *LY6K* promoter (Figure 5A). Although both active and inactive marks of chromatin are present throughout the LY6K promoter region, H3K4me3 active marks were enriched especially in the −250 and −100 CGI shore region of the LY6K in MCF7-ADR cells, which highly express LY6K compared with that of the LY6K-negative/low expressing cell lines. Conversely, H3K27me3 inactive marks were depleted compared with that in the LY6K-negative cell lines. However, acH3K9 and H3K9me3 marks did not show much of a difference between the LY6K-positive and LY6K-negative cell lines than the difference observed for trimethylation of H3K4 and H3K27. Furthermore, we performed MSP analysis using bisulfite-modified ChIP DNA samples to examine how the active and inactive chromatin marks were distributed with respect to the DNA methylation patterns of *LY6K* in the −350 to +100 region (Figure 5B). The ChIP-MSP analysis in the MCF7-ADR cell line showed that acetyl-H3K9 and trimethyl-H3K4 were predominantly associated with alleles presenting unmethylated DNA. Trimethyl-H3K9 and trimethyl-H3K27 were generally associated with methylated DNA, but this pattern was less distinct than the active marks (Figure 5B).

## DISCUSSION

The human LY6K gene is aberrantly expressed in various carcinomas and has been identified as a target antigen for diagnosis and cancer vaccine therapies. LY6K is upregulated in breast cancer tissues compared with NTL expression [14]. Moreover, it increases invasion and migration of cancerous cells by activating the ERK signaling pathway in breast cancer cell lines [18]. These findings suggest that LY6K could be associated with tumor progression and metastasis. In our study, we found that patients with high levels of LY6K had an aggressive, metastasizing tumors regardless of treatment with adjuvant chemotherapy or endocrine therapy, and ER-positivity of tumors. In addition, knockdown of LY6K delayed tumor progression in secondary xenograft tumors. These results suggest that LY6K is an independent prognostic marker of breast cancer and metastasis.

EMT is known to be a key process in tumor invasion, metastasis, and tumorigenicity. Several EMT-regulating markers, including E-cadherin, SNAI1, ZEB1, and vimentin are involved in this process [25]. EMT is also involved in epithelial breast cancer metastasis, and during this transition, cells lose their epithelial characteristics and acquire mesenchymal properties [26]. Here, we found that LY6K downregulates the expression of E-cadherin in breast cancer cells, carcinomas and xenograft mice, whereas does not increase vimentin. Loss of E-cadherin is considered a fundamental event in EMT, thus our finding suggests that LY6K activates EMT initiation, inducing metastasis. Together, these data indicate that LY6K functions as an upstream component of the metastasis signaling network that can suppress E-cadherin expression at the transcription level.

Chromatin structure, including DNA methylation, histone variants and modifications, and nucleosome positioning as well as non-coding regulatory RNAs are important in the cancer epigenetic pathway [27]. Interestingly, only about 70% of human genes contain a CGI promoter and only 6.8% of CpGs reside within CGIs, many potentially informative CpG sites remain to be examined [28, 29]. A recent study showed that DNA methylation directly silences genes with non-CGI promoters and contributes to establishing tissue-specific methylation patterns [30]. Furthermore, tissue- and cancer-specific differentially methylated regions occur more frequently within CGI shores than within CGIs themselves, suggesting the involvement of CGI shore in methylation during tissue differentiation, epigenetic reprogramming and cancer [31, 32]. In this study, LY6K gene expression was altered by DNA methylation of not only the LY6K CGI but also non-CGI promoter, the CGI shore. Our data suggested that methylation pattern of LY6K is useful biomarker of cancer because LY6K expression was cancer-specifically decreased by CGI shore methylation.

Interestingly, LY6K expression was lower in the SNP242 mutation which creates *de novo* PAX3 transcription factor binding sites in breast cancer cell lines [23]. PAX3 reported as a key factor for normal development and tumorigenesis, and a transcription repressor that binds with HP1 and KAP1, which closed chromatin [24]. We found that PAX3 binding sites, created by SNP242, differentially increase LY6K promoter methylation, which in turn influences migration of breast cancer cells [23]. Moreover, a potential correlation between DNA methylation status and SNP242 status was found in breast cancer cell lines [23]. Based on these findings, we can predict LY6K methylation status by determining SNP242 status. Taken together, our results indicate that LY6K aberrant expression results from the aberrant methylation of LY6K promoter, induced by *de novo* PAX3 binding sites.

Additionally, LY6K gene expression was activated or inhibited by trimethylation of H3K4 or H3K27 histone modification around the transcription start site, respectively. These results suggest that H3K4 trimethylation may be important in determining LY6K expression, whereas H3K27 trimethylation may be important for LY6K repression. This analysis confirmed that active chromatin marks are associated with unmethylated DNA, whereas inactive chromatin marks are associated with methylated DNA. Taken together, our data indicate that the epigenetic activation, determined by the presence of active chromatin marks on unmethylated DNA, allows LY6K continued expression.

**Figure 6:**
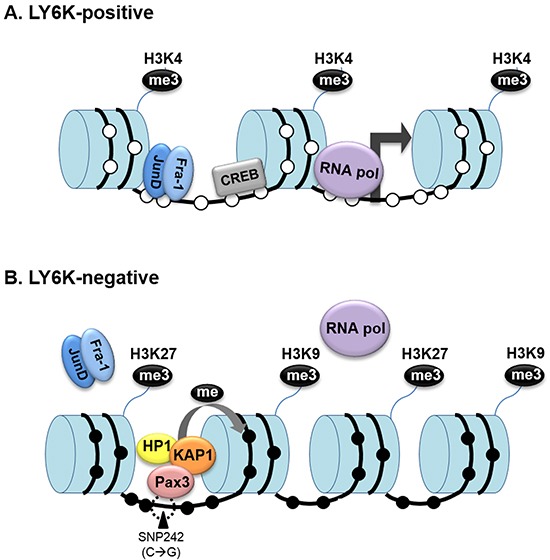
Dynamic epigenetic modification of LY6K influences the LY6K gene activity **A.** In LY6K-positive cells, methylation of the histone H3 tail, lysine 4, and demethylation of DNA, which contribute to a more open chromatin conformation that allows RNA polymerase II (Pol II) to bind to the LY6K promoter. **B.** Because of the presence of trimethylated histone K9 and K27 and methylated DNA caused by SNP242 mutation, chromatin is in a closed conformation in LY6K negative cells that blocks access to key transcription factors.

Subsequently, we confirmed the relationship between DNA methylation and histone modification within the LY6K CGI and CGI shore. In particular, the chromatin was open in the functional LY6K promoter region, LY6K CGI shore, which is essential for gene activation. In addition, the CpG site within this region was hypomethylated. These observations indicate that the AP-1 transcription factor preferentially binds to the LY6K promoter and can activate the gene (Figure 6). The CpG site within the closed chromatin region showed a hypermethylated pattern, suggesting that LY6K gene expression in cancer can be predicted using DNA methylation status and a chromatin mark in the functional 5′CGI shore of LY6K (Figure 6). These results suggest that aforementioned epigenetic features of LY6K might be specific to breast tissue and breast cancer. Taken together, epigenetic alterations, particularly DNA methylation of the LY6K CGI shore and CGI, may contribute to LY6K activation in breast cancer.

In this study, we identified and validated in both cellular models and clinical samples that LY6K is associated with the presence of distant metastasis and patient survival. In addition, knockdown of LY6K delayed tumor progression in xenograft tumors. We further showed that LY6K aberrant expression is the result of DNA methylation and histone modifications in LY6K CGI shore and CGI. We elucidated that PAX3 binds to LY6K promoter binding sites created by SNP242 and suppresses LY6K expression through the regulation of DNA methylation.

In conclusion, our findings show that LY6K is frequently activated by CGI shore hypomethylation and active histone marks in breast carcinomas. High levels of LY6K expression induce a more aggressive and metastatic phenotype. In addition, LY6K methylation might be an independent prognostic marker in breast carcinoma and may be useful for selecting patients with higher risk of recurrence, death, and metastasis and for chemotherapy response. Targeting LY6K methylation might be a potential strategy for treating patients with hypomethylated tumors, although further investigation in this area is needed.

## MATERIALS AND METHODS

### Patients and samples

The original microscopic slides, paraffin-embedded tissue samples, and clinical information from 144 female patients with breast carcinoma diagnosed between January 1997 and August 2002 were obtained for this study. The patients underwent the modified radical mastectomy or breast conserving surgery in Chonbuk National University Hospital. Among the 144 patients, 120 patients received post-operative endocrine therapy, 128 patients received adjuvant chemotherapy, and 109 patients received both endocrine therapy and chemotherapy. The pathologic features and tumor stage were reviewed by two pathologists (Jang KY and Kim KM) according to the World Health Organization Classification [20] and the 7^th^ edition of the American Joint Committee on Cancer staging system [21]. This study was approved by the Institutional Reviewer Board of Chonbuk National University Hospital (IRB number: 2013-08-016). Informed consent was provided according to the Declaration of Helsinki.

### Immunohistochemical staining and immunohistochemical scoring for tissue microarrays

Immunohistochemical staining for LY6K (1:250, Sigma-Aldrich, St. Louis, MO, USA) was performed on tissue microarray blocks with 3.0 mm sized core. Briefly, one core per case was arrayed in a tissue microarray. The antigen retrieval procedure was performed with a microwave oven for 20 min in pH 6.0 sodium citrate buffer. The scoring for LY6K immunostaining was performed by two pathologists (Jang KY and Kim KM) by consensus without knowledge of the clinicopathological information. LY6K immunostaining was evaluated to estimate the positivity of tumor cells. The staining intensity was scored as 0 (no staining), 1 (weak staining), 2 (moderate staining), and 3 (strong staining). The area of staining was evaluated as 0 (no stained cells), 1 (1–10% of the cells stained positive), 2 (11–33% of the cells stained positive), 3 (34–66% of the cells stained positive), and 4 (67–100% of the cells stained positive). Thereafter, the sum of intensity score and proportion score was used for further analysis. The maximum sum score was seven and the minimum sum score was zero.

### Cell culture and treatment

Human breast carcinoma MCF7/ADR cell was provided by Dr. YM Park (Roswell Park Cancer Institute, Buffalo). And MCF7, MDA-MB-231, MDA-MB-435s, MDA-MB-453, MDA-MB-468, SK-BR-3 cells were obtained from Sapporo Medical University. These cell lines were tested using short tandem repeat markers for DNA fingerprinting analysis (Korean Cell Line Bank). Other human breast carcinoma T47D, ZR-75-1, MDA-MB-157, MDA-MB-361, and MDA-MB-436 cells were purchased from ATCC (Manassas, VA). Mouse adapted MDA-MB-231 cells were obtained from primary tumor of MDA-MB-231 xenograft mice. Breast cancer cells and MDA-MB231/A were grown in Dulbecco's Modified Eagle's Medium (WelGENE Inc., Deajeon, Korea) containing 10% fetal bovine serum (WelGENE) and maintained at 37°C in a humidified atmosphere with 5% CO_2_ and 95% air. Cells were seeded at 5 × 10^5^ cells on 60 mm dishes 24 h prior to treatment. The cells were treated every day with 2 or 5 μM 5-aza-2′-deoxycytidin (5-aza-dC; Sigma-Aldrich, St. Louis, MO, USA) for 72 h to block CpG methylation and/or followed by treatment with 100 nM trichostatin A (TSA; Sigma) for 6 h to inhibit histone deacetylation.

### Establishment of stable cell lines expressing siLY6K

The piLenti-siLY6K (LV014) and lentiviral packaging plasmids (LV003) were purchased from Applied Biological Materials, Inc (abm^®^) and cotransfected into HEK293T cells following the manufacturer's protocol using LentiFectin transfection reagent (G074). The lentiviruses in the supernatant was collected, centrifuged and used to infect mouse adapted MDA-MB-231 cells. After puromycin selection for 2 to 4 weeks, stable clones were obtained and subsequently confirmed by qRT-PCR and western blotting.

### Preparation of breast cancer xenograft mice

All studies involving the use of nude mice were approved by the Animal Care and Use Committee of Yonsei University Medical School (2014-0348) and performed in specific pathogen-free facilities and under conditions in accordance with the Guidelines for the Care and Use of Laboratory Animals of YUMS. Mice were inoculated subcutaneously with 1 × 10^6^ stable MDA MB-231-(vector), MDA MB-231-(shLY6K) cells into each flank under 150 μL of saline/zoletil/rompun (7:1:1) anesthesia. Mice were randomized into groups (n = 5 per group). From palpable tumor formation until termination, tumor sizes were measured every 2 to 3 days using calipers, and tumor volume was calculated with the following formula: length × width^2^ × 0.5236. Mice were sacrificed in a 7.5% CO_2_ chamber, and tumors were harvested for immunohistochemical and other analyses.

### Total RNA isolation and qRT-PCR

Cellular RNA was isolated from breast cancer cell lines using a NucleoSpin® DNA/RNA/Protein kit (MACHEREY-NAGEL, Düren, Germany), according to the manufacturer's instructions. RNA (2–5 μg) was reverse transcribed to cDNA using the M-MLV Reverse Transcription kit (Promega, Madison, WI, USA). About 1 μL aliquot of cDNA was used as the template to amplify a specific fragment in a 30 μL reaction mixture under the following conditions: denaturation at 94°C for 5 min, 30 cycles at 94°C for 30 s, 58°C for 30 s and 72°C for 30 s, and then an extension at 72°C for 5 min. And qRT-PCRs were performed with LightCycler 96 SYBR Green I Master mix (Roche, Basel, Switzerland) on an ABI Real-time PCR 7500 system using human 18s rRNA as endogenous control. All reactions were performed in triplicate.

### DNA isolation and bisulfite treatment

Genomic DNA was extracted from breast cancer cell lines using a NucleoSpin® total DNA/RNA/Protein kit (MACHEREY-NAGEL), following the manufacturer's instructions. Unmethylated cytosine bases of genomic DNA (500 ng) were converted to uracil using the EZ DNA Methylation-Gold kit (Zymo Research, San Francisco, CA, USA).

### Bisulfite pyrosequencing

Sequences after bisulfite treatment were analyzed using Pyrosequencing™ (PyroMark ID System, Biotage, Stockholm, Sweden) technology. Primers selected for amplification were designed using PyroQ-CpG™ Software (Biotage) and listed in Supplementary Table S1. Amplification was performed using 100 ng of total DNA in a 50 μL PCR reaction mix. Samples were denatured for 5 min at 94°C followed by 44 cycles of amplification at 94°C for 30 s, 55°C for 1 min, and then 72°C for 90 s. A 30 μL aliquot of PCR product was used for immobilization by streptavidin sepharose beads (Streptavidin Sepharose™ high performance, GE Healthcare Bio-Science AB, Uppsala, Sweden) and the single-stranded DNA was incubated with the biotinylated sequencing primer at 95°C for 10 min.

### Melting curve analysis (MCA)

Methylated and unmethylated DNA was purchased from Qiagen (Valencia, CA, USA) as a positive and negative control respectively. Methylation standards (100%, 75%, 50%, 25%, and 0%) were prepared by mixing the positive and negative controls accordingly. Primers used for MCA were designed using the Sequenom (http://www.epidesigner.com) listed in Supplementary Table S1. Bisulfite converted DNA was amplified using the MCA primer sets with LightCycler 96 SW 1.1 (Roche) in the presence of LightCycler 96 SYBR Green I Master (Roche). The PCR reactions were cycled for 45 cycles of at 94°C for 30 s, 54°C for 30 s and 72°C for 30 s, and then an extension at 72°C for 3 min. After the amplification, temperature was gradually increased from 65 to 95°C to obtain the melting curves. The melting curve peaks were plotted by using the LightCycler 96 software (Roche) and by calculating the negative derivative of fluorescence over temperature. The area under the curve (AUC) was quantified for the melting peak corresponding to the unmethylated and methylated alleles. In addition, the percentage of DNA methylation was estimated on the basis of the linear regression between the AUC values and the methylation standards (Supplementary Figure S4).

### Methylation-specific PCR (MSP)

Primers specific for LY6K unmethylated and methylated alleles were used to amplify bisulfite-treated DNA. Methylation specific PCR primers were designed for the LY6K promoter and for parts of *LY6K* gene body, from −300 to +1000. The primer sequences are shown in Supplementary Table S1 and their location is presented in Supplementary Figure S4. MSP was conducted with 1 μL bisulfite-modified genomic DNA that was mixed with 10× MSP buffer (166 mM (NH_4_)_2_SO4, 670 mM Tris (pH 8.8), 67 mM MgCl_2_ and 100 mM 2-mercaptoethanol), 25 mM dNTP, MSP forward and reverse primers, and HotStar Taq® *Plus* DNA polymerase (Qiagen) using the following cycling conditions: denaturation at 94°C for 15 min, followed by 35 cycles at 94°C for 30 s, 55°C for 30 s, and 72°C for 1 min, with a final extension at 72°C for 5 min. The MSP bands were detected on 2% agarose gel electrophoresis.

### Western blotting

Proteins extracts were prepared using a NucleoSpin® DNA/RNA/Protein kit (MACHEREY-NAGEL), following the manufacturer's protocol. Immunoblotting was carried out according to a previously described method using anti-LY6K (I-14; Santa Cruz Biotechnology, Santa Cruz, CA, USA) and anti-β-actin (Bethyl Laboratories, Inc. Montgomery, TX, USA) antibodies [23].

### Chromatin immunoprecipitation (ChIP) assay

The ChIP assay was performed using the imprint chromatin immunoprecipitation kit (CHP1, Sigma-Aldrich) following the manufacturer's instructions. The sonicated DNA was incubated with anti-IgG (Millipore, Milford, MA, USA) as a negative control, anti- H3K9ac (Millipore), anti-H3K4me (Millipore), anti-H3K9me3 (Abcam, Cambridge, MA, USA) and anti-H3K27me3 (Millipore) at 37°C for 90 min. The purified DNA was amplified using specifically designed real-time ChIP PCR primers spanning the region −300 to +1000 of *LY6K* (Supplementary Table S1).

### ChIP-MSP

Approximately 25 μL of the 50 μL ChIP DNA product was bisulfite modified as described above. MSP was performed using the *LY6K* MSP primers listed in Supplementary Table S1.

### Statistical analysis

LY6K immunohistochemical expression was grouped as negative, low-expression, or high-expression by receiver operating characteristic curve analysis for the prediction of the risk of death (Supplementary Figure S1). The cut-off point between high-LY6K and low-LY6K was seven and the cut-off point between low-LY6K and LY6K-negative was four (LY6K-negative, score 0–3; low-LY6K, score 4–6; high-LY6K, score 7). To compare the association between clinicopathologic variables and LY6K expression, Pearson's chi-square test were used. The prognostic significance of the LY6K expression in patients with breast carcinoma was analyzed by evaluating OS, event-free survival (EFS), and distant metastatic relapse (DMR). The follow-up end point was the date of the last contact or the date of death through December 2012. Cox proportional hazards regression analyses and Kaplan-Meier survival analysis were performed using SPSS software (version 19.0, SPSS, Chicago, IL, USA). Student's *t*-tests were used to evaluate differences using GraphPad Prism 5 Software (GraphPad Software Inc., San Diego, CA, USA). The non-parametric, Pearson's and Spearman's rank test, was used to examine the correlation between expression and methylation of LY6K. *P* values < 0.05 were considered statistically significant (n = 3 for each experiment).

## SUPPLEMENTARY FIGURES AND TABLES


