# Supplementary material for: Epigenetic activation of LY6K predicts the presence of metastasis and poor prognosis in breast carcinoma
Source: Oncotarget. 2016 Aug 1;7(34):55677–89. doi: 10.18632/oncotarget.10972 (PMC5342445; doi:10.18632/oncotarget.10972)
Supplement: Supplementary file 1 [file oncotarget-07-55677-s001.pdf]

Epigenetic activation of LY6K predicts the presence of metastasis and poor prognosis in breast carcinoma

SUPPLEMENTARY FIGURES AND TABLES

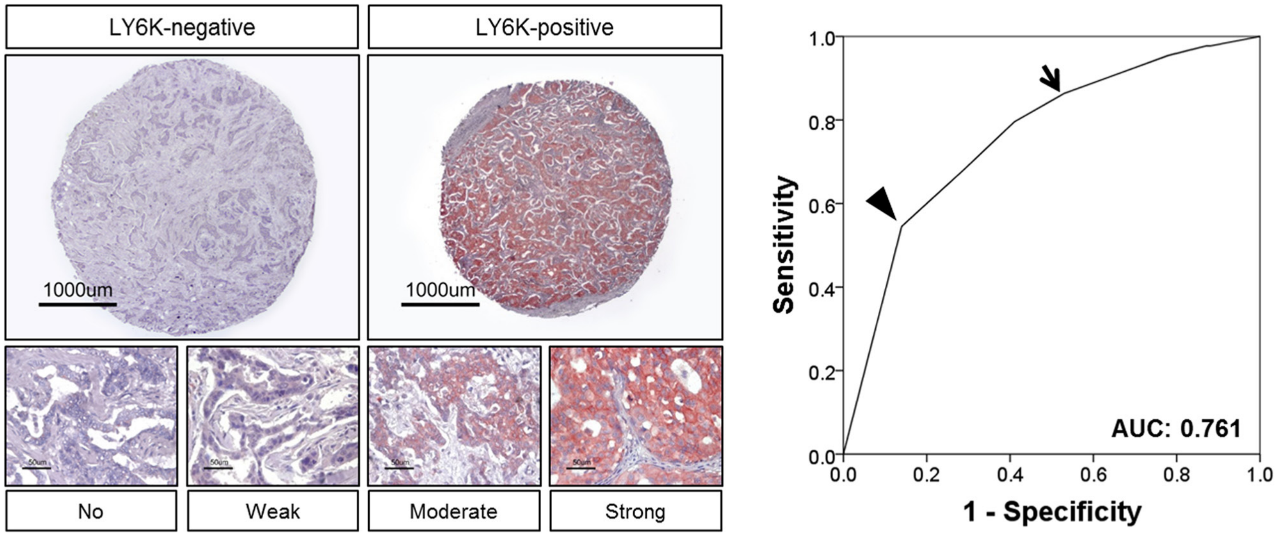

Supplementary Figure S1: Immunohistochemical expression of LY6K and analysis of sensitivity and specificity of LY6K immunohistochemical scores for the death of the patient by receiver operator characteristic curve. Arrow head indicates a cut-off point between specimens with high-LY6K and low-LY6K and arrow indicates a cut-off point between specimen with low-LY6K and LY6K-negative.

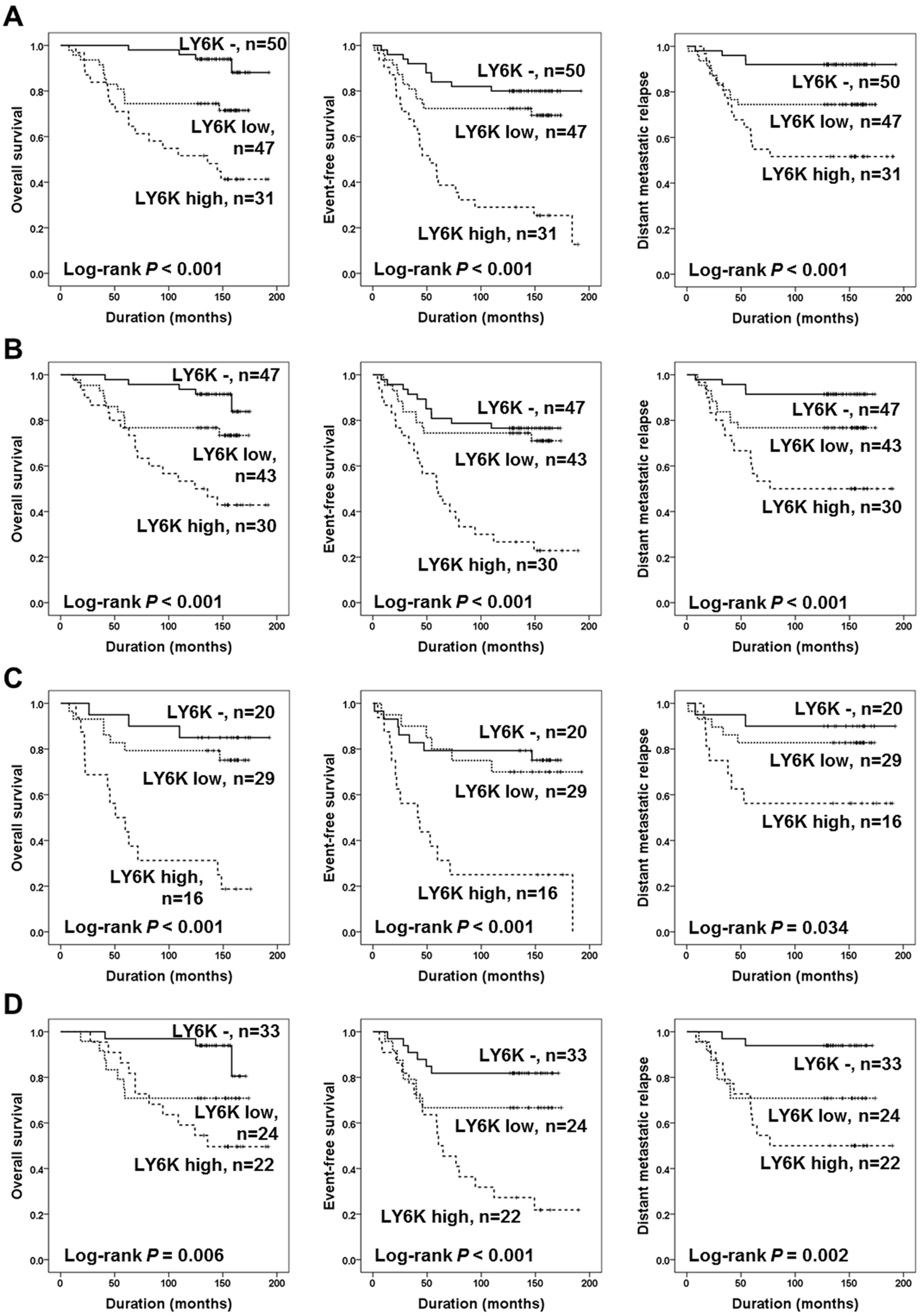

Supplementary Figure S2: Kaplan-Meier survival analysis in variable subgroups of patients with breast carcinoma. Overall survival, event-free survival, and distant metastatic relapse according to the LY6K expression in **A.** 128 patients with breast carcinoma who received adjuvant chemotherapy **B.** 120 patients who received post-operative endocrine therapy **C.** 65 patients with estrogen receptor negative tumors and **D.** 79 patients with estrogen receptor positive tumors.

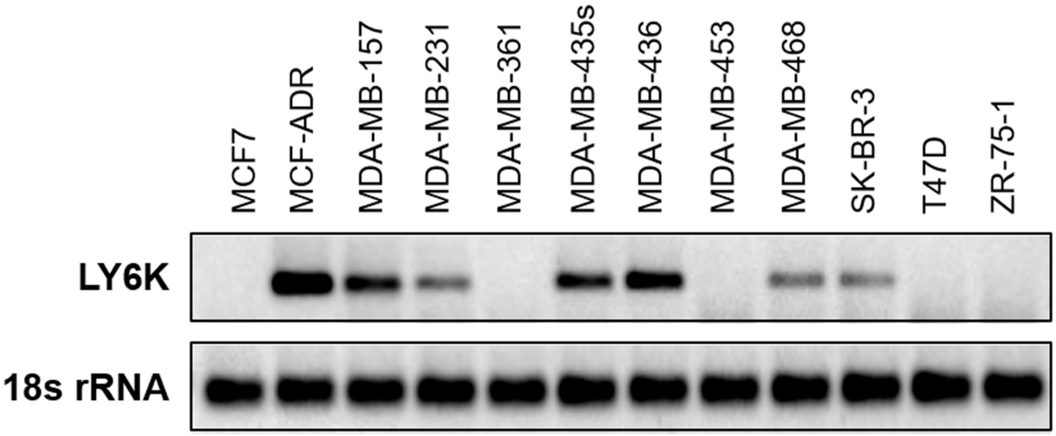

Supplementary Figure S3: Analysis of LY6K expression in breast cancer cell lines. Basal expression of LY6K transcripts in breast cancercell lines.

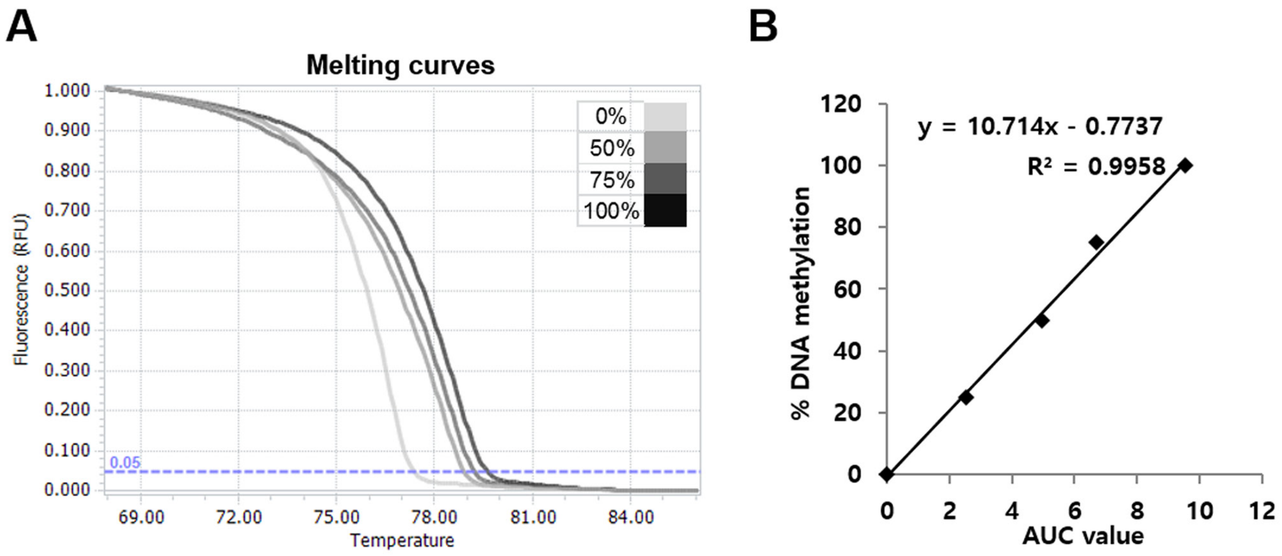

Supplementary Figure S4: Assessment of LY6K methylation by Melting Curve Analysis (MCA). A. Melting curves and melting peakscorresponding to 100%, 75%, 50% and 0% methylation standards areshown. **B.** Linear regression between the AUC values of eachmethylation standards and their corresponding percentage of methylation.The AUC on the methylated peaks was used to estimate the level of DNA methylation in breast cancer cell lines and tumors.

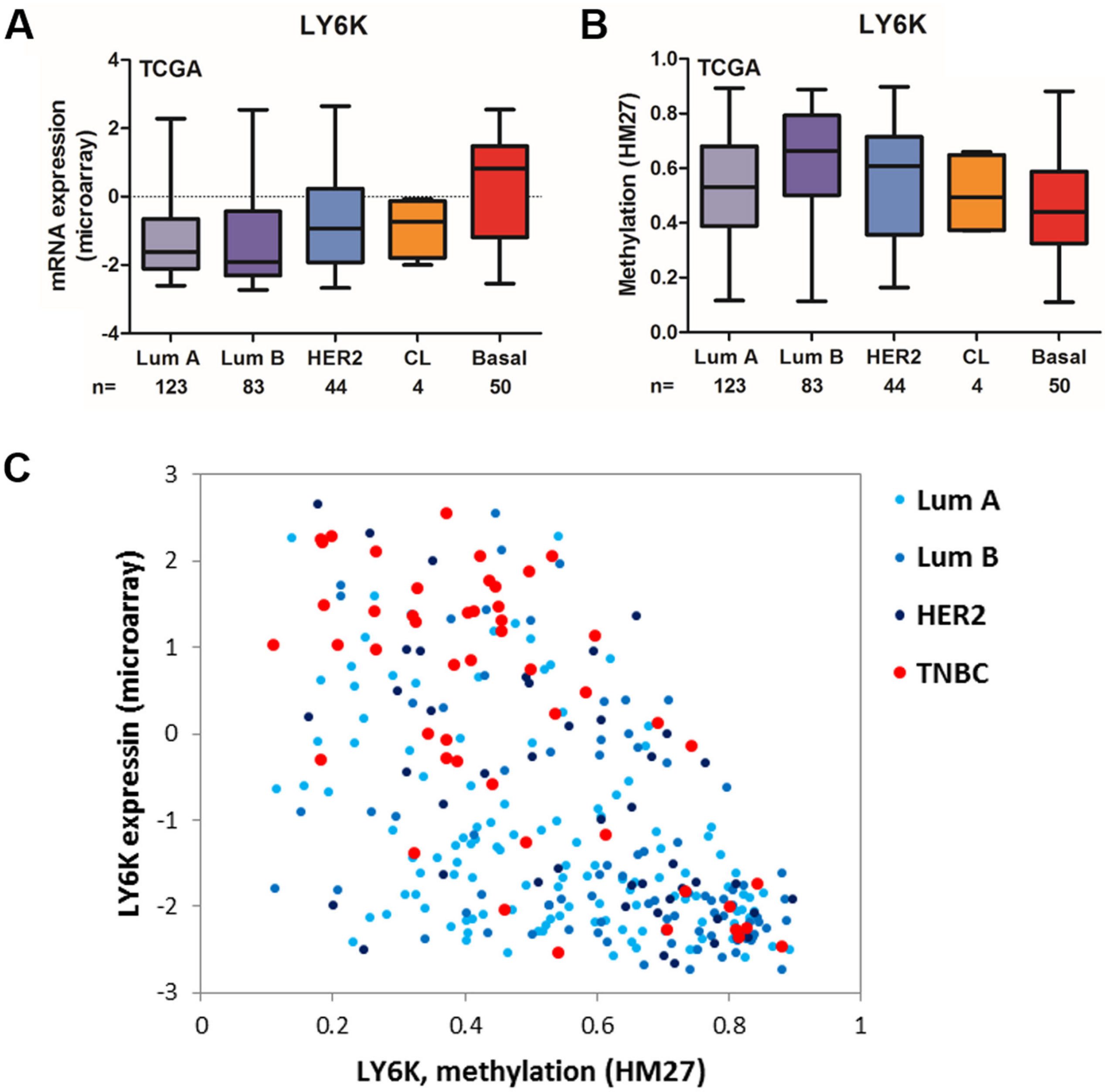

Supplementary Figure S5: LY6K expression negatively correlated with methylation of LY6K. A. mRNA microarray and **B.** methylation chip (HM27) of LY6K analyzed in TCGA data base. **C.** The inverse correlation of LY6K expression and methylation.

Supplementary Table S1: Specific primers for PCR and PCR amplification of MSP, bisulfite-pyrosequencing, bisulfite sequencing, and ChIP assay

Primer	Sense	Antisense
LY6K	TGCTCGCCTTGCTGCTGGTC	TCGCTGCACAACCAGCGGAG
CDH1	TGCCCAGAAAATGAAAAAGG	GTGTATGTGGCAATGCGTTC
Vimentin	CCCTCACCTGTGAAGTGGAT	GCTTCAACGGCAAAGTTCTC
Pax3	AGTGAGATTACGCGCTAG	CCAGCGGAAGACCAGAAAC
18s rRNA	GTAACCCGTTGAACCCCATT	CCATCCAATCGGTAGTAGCG
MCA	GTTTGAAAGAGGAATTGTTTAAAAG	TTACTAATAAAACCCCTACCTAAACC
MSP1 U	TTGTTTAAAAGAATGAGTTAGTTGT	CCCTAAAAACTTCCCAAAACATA
MSP1 M	TTGTTTAAAAGAATGAGTTAGTCGT	CCTAAAAACTTCCCAAAACGTA
MSP2 U	AGTGGAGAAGATTTTTTATTTGGTT	CCCACCTTATACTACTATTAAAACAAC
MSP2 M	CGGAGAAGATTTTTTATTTGGTC	CCCGCCTTATACTACTATTAAAACG
BSP1	TGTGGTTTAGGTTGGAGTGTAGTG	AAAAAAAAAAACAAAAACCACATC
BSP2	TGGAATTTTTTTTTAGGGATGTG	TCTTTAAAACCTCATCTCAAAACC
CHIP1	CCCGGGTTCACGCCATTCTCCT	TTCCTCCTGGCGCCTGTAGTC
CHIP2	CAGGAGGAATTCTTATTTGAGC	CCCCTGCCTGGGCCGTTCGTGA
CHIP3	CCGCCGGCCACTTTCTGT	AGTCCCGCCTTGTGCTGCTGTT
CHIP4	GAGGCTCCAAAGACCCCGACAG	CGCCCCCTCCCCGCCCTTCT

Supplementary Table S2: The association between clinicopathologic variables and the expression of LY6K

Characteristics		# of cases	LY6K			*p*-value
			negative	low	high	
Age, y	< 50	103	44 (43%)	39 (38%)	20 (10%)	0.006
	≥ 50	41	9 (22%)	14 (34%)	18 (44%)	
TNM stage	I	29	13 (45%)	10 (34%)	6 (21%)	0.852
	II	96	6 (6%)	8 (8%)	5 (5%)	
	III and IV	19	6 (32%)	8 (42%)	5 (26%)	
T stage	1	41	17 (41%)	15 (37%)	9 (22%)	0.324
	2	94	34 (36%)	32 (34%)	28 (30%)	
	3 and 4	9	2 (22%)	6 (67%)	1 (11%)	
LN metastasis	absence	82	36 (44%)	25 (30%)	21 (26%)	0.095
	presence	62	17 (27%)	28 (45%)	17 (27%)	
Latent distant metastasis	absence	112	49 (44%)	41 (37%)	22 (20%)	< 0.001
	presence	32	4 (13%)	12 (38%)	16 (50%)	
Histologic type	ductal	136	53 (39%)	49 (36%)	34 (25%)	0.07
	lobular	8	0 (0%)	4 (50%)	4 (50%)	
Histologic grade	1	48	24 (50%)	14 (29%)	10 (21%)	0.068
	2	67	24 (36%)	24 (36%)	19 (28%)	
	3	29	5 (17%)	15 (32%)	9 (31%)	
HER2	negative	105	44 (42%)	37 (35%)	24 (23%)	0.089
	positive	39	9 (23%)	16 (41%)	14 (36%)	
ER	negative	65	20 (31%)	29 (45%)	16 (25%)	0.194
	positive	79	33 (42%)	24 (30%)	22 (28%)	
PR	negative	60	19 (32%)	24 (40%)	17 (28%)	0.557
	positive	84	34 (40%)	29 (35%)	21 (25%)	

Abbreviations: LN, lymph node; HER2, human epidermal growth factor receptor 2; ER, estrogen receptor; PR, progesterone receptor.

Supplementary Table S3: Univariate Cox regression analysis for overall survival and event-free survival in breast carcinoma patients

Characteristics		N	OS		EFS		DMR	
			HR (95% CI)	*p*-value	HR (95% CI)	*p*-value	HR (95% CI)	*p*-value
Age, y	< 50	103	1	< 0.001	1	0.021	1	0.029
	≥ 50	41	2.893 (1.600-5.230)		1.866 (1.098-3.170)		2.127 (1.080-4.189)	
TNM stage	I	29	1	0.016	1	0.312	1	0.053
	II	96	2.515 (0.886-7.142)	0.083	1.384 (0.669-2.865)	0.381	1.827 (0.630-5.303)	0.267
	III and IV	19	5.156 (1.615-16.462)	0.006	2.016 (0.816-4.982)	0.129	3.984 (1.199-13.242)	0.024
Histologic grade	1	49	1	0.016	1	0.136	1	0.101
	2	66	1.187 (0.561-2.514)	0.654	1.190 (0.634-2.233)	0.589	1.105 (0.478-2.553)	0.816
	3	29	2.747 (1.261-5.985)	0.011	1.966 (0.979-3.950)	0.058	2.318 (0.960-5.596)	0.061
HER2	negative	105	1	< 0.001	1	0.001	1	0.023
	positive	39	2.879 (1.588-5.220)		2.396 (1.408-4.077)		2.216 (1.118-4.392)	
ER	negative	65	1	0.242	1	0.922	1	0.691
	positive	79	0.072 (0.388-1.270)		0.974 (0.578-1.643)		1.149 (0.580-2.274)	
PR	negative	60	1	0.075	1	0.091	1	0.441
	positive	84	0.584 (0.323-1.056)		0.637 (0.377-1.075)		0.767 (0.391-1.504)	
LY6K	negative	53	1	< 0.001	1	< 0.001	1	< 0.001
	low	53	2.626 (1.009-6.836)	0.048	1.352 (0.633-2.890)	0.436	3.378 (1.089-10.477)	0.035
	high	38	7.345 (2.997-18.002)	< 0.001	4.864 (2.479-9.543)	< 0.001	7.711 (2.607-22.808)	< 0.001

Abbreviations: OS, overall survival; EFS, event-free survival; DMR, distant metastatic relapse; HR, hazard ratio; 95% CI, 95% confidence interval; HER2, human epidermal growth factor receptor 2; ER, estrogen receptor; PR, progesterone receptor.
